# Knowledge and experiences of overdose among formerly incarcerated people living with HIV and hazardous opioid use in South Africa: A qualitative study

**DOI:** 10.1371/journal.pone.0361907

**Published:** 2026-11-02

**Authors:** Urvisha Bhoora, Yangxi An, Sarah Pollock, Pretty Ndini, Derrick Moyo, Tonderai Mabuto, Laura Steiner, Jill Owczarzak, Christopher J. Hoffmann

**Affiliations:** 1 Department of Family Medicine, University of Pretoria, Tshwane, South Africa; 2 Community Oriented Primary Care Research Unit, Department of Family Medicine, University of Pretoria, Tshwane, South Africa; 3 Department of Public Health Studies, Krieger School for the Arts and Sciences, Johns Hopkins University, Baltimore, United States of America; 4 Department of Health, Behavior and Society, Johns Hopkins University Bloomberg School of Public Health, Baltimore, United States of America; 5 Implementation Research Division, Aurum Institute, Johannesburg, South Africa; 6 Department of Medicine, Johns Hopkins University School of Medicine, Baltimore, United States of America; University of Toronto, CANADA

## Abstract

**Background:**

Drug overdose is a leading cause of death among people released from correctional facilities, and the risk of overdose is higher among people living with HIV (PLWH). Overdose remains understudied in people in South Africa despite the high burden of HIV and hazardous opioid use. The researchers aimed to qualitatively explore how formerly incarcerated people living with HIV and OUD in South Africa understand, experience, and respond to overdose.

**Methods:**

People with HIV and self-reported hazardous opioid use prior to correctional facility release or shortly after release were enrolled in a convenience sample. Twenty-three formerly incarcerated people living with HIV and opioid use in or around Tshwane, South Africa completed sequential in-depth interviews that explored medication-assisted treatment for hazardous opioid use and HIV care delivery. Transcripts were analyzed thematically using both deductive and inductive coding, with a focus on participants’ knowledge and definition of overdose, personal experiences with overdose, and responses to overdose.

**Results:**

All participants used the term “overdose”, but there was no unified understanding of what constitutes an overdose or how to manage an overdose. The period immediately following release from incarceration was identified as a key moment in which overdose occurred. Pouring water onto the person overdosing was reported to be the most prevalent response strategy. Only two participants contacted or watched their peers contact the ambulance. Fear of law enforcement involvement was perceived as a barrier to calling for emergency services.

**Conclusion:**

The findings identify a gap in overdose knowledge among corrections-involved people living with HIV and hazardous opioid use in South Africa. There is a need to increase knowledge regarding overdose recognition and responses.

## Background

Opioid use has been increasing over the last decade in sub-Saharan Africa, including in South Africa [1]. Heroin, known locally in South Africa as *nyaope or whoonga*, has contributed to a surge in opioid-related problems, especially among marginalized population groups [2,3]. With an average price of 25–30 rands (1.33 to 1.59 United States (U.S.) dollars), *nyaope* is highly accessible to low-income communities, and is used through smoking or injection [4]. Opioid overdoses are a potentially fatal consequence of opioid use. In high-income countries such as the U.S., opioids are involved in 75% of drug overdose deaths [5,6]. In a small study conducted among people who use drugs in Cape Town, Durban, and Pretoria, 38% of participants self-reported an opioid overdose in the past year although understanding of overdose and symptoms at the time of overdose were not assessed [7].

People released from correctional facilities are at a substantially higher risk of overdose and overdose mortality than people who use opioids overall, with the risk increasing in people living with HIV (PLWH) [8–12].This risk is highest during the first few weeks after release from incarceration and remains elevated up to at least the fourth week [13]. Factors that contribute to an elevated risk include a decrease in drug tolerance during incarceration due to less drug access or access to lower purity drugs, interruptions in medical care on release, and disruptions in social support when returning to the community that may lead to increased unobserved substance use [14].

Overdose and overdose deaths are preventable through overdose education – including recognizing the signs of an overdose – and through community access to opioid antagonists. Given the heightened risk of overdose during community re-entry following incarceration, and the large numbers of incarcerated people with hazardous opioid use in South Africa, and globally, this population represents a critical target for overdose intervention. In South Africa, an estimated 23% to 42% of the incarcerated population reports substance use [15–17]. Although robust population-level data on the prevalence of opioid use disorder within correctional facilities remain limited. The intersection of opioid use, HIV, TB and incarceration represents an important public health concern, given the high burden of HIV and TB in South African correctional settings [18,19]. Daily opioid use disorder has been documented among 22% of people living with HIV being released from incarceration in Gauteng Province, South Africa [18]; in a similar geography, the prevalence of HIV has been measured as 35% among people who use drugs [19,20]. Despite this elevated risk, there are few descriptions of overdose knowledge and experiences with overdose among people with hazardous opioid use in South Africa. Understanding how individuals with lived experiences perceive, interpret, and respond to overdose is essential to informing relevant prevention and treatment strategies. We therefore sought to explore: (1) how people recently released from incarceration and living with HIV and hazardous opioid use understand overdose; (2) their knowledge of overdose symptoms and risk, and (3) their approaches to responding to an overdose event.

## Methods

### Study site

This study was conducted in the Kgosi Mampuru II Correctional Management Area and in the Tshwane district, both located in Gauteng Province, South Africa. Kgosi Mampuru II is a correctional facility that typically houses an average of 7000–8000 individuals. While the facility houses individuals from around the country, many return to communities in the Tshwane district. This facility was selected due to the size and proximity to Tshwane, a region known to have a substantial number of people who use substances [20].

### Study design

The researchers conducted this qualitative study as part of a broader mixed methods single-arm cohort study focused on characterizing medication-assisted treatment for hazardous opioid use and HIV care delivery for people returning to the community in South Africa [19,21]. Qualitative in-depth interviews with formerly incarcerated individuals living with HIV and current or past opioid use who resided in or close to Tshwane, South Africa, were conducted. The participants were introduced to and received harm reduction services at the Community Oriented Substance Use Programme (COSUP), a program implemented by the University of Pretoria in the City of Tshwane Metropolitan Municipality.

### Study participants

A convenience sample of participants was prospectively enrolled in the parent study. Eligible participants were: (1) either incarcerated with an imminent expected release date within 90 days or formerly incarcerated with a recent release date (90 days); (2) ≥ 18 years; (3) self-reported current or previous hazardous use of illicit opioids; (4) living with HIV; (5) expected release residence in Tshwane; and (6) able to provide informed consent. We have used the words hazardous opioid use rather than opioid use disorder because enrollment was not based on a clinical assessment but rather on self-reported opioid use, prior efforts to stop using, and reported negative impact on health, responsibilities, or interpersonal relationships. Recruitment occurred in both the correctional facility and in the community. In the correctional setting, study team members delivered presentations to inform people who were incarcerated of the study, and potential study participants with anticipated release were referred by correctional facility health staff for eligibility screening. In the community setting, parole officers referred individuals who had been recently released for eligibility screening. No information on enrollment was returned to the correctional facility health staff or parole officers. This study was completed in compliance with the principles of the Declaration of Helsinki. All participants provided written informed consent. Ethical approval was provided by the University of the Witwatersrand Human Research Ethics Committee, the University of Pretoria Human Research Ethics Committee, the South African Department of Correctional Services, and the Johns Hopkins University School of Medicine Institutional Review Board.

### Data collection

Participants completed an individual baseline interview with a study team member. Basic socio-demographic data and location information were collected. Within one week of either release from the correctional facility or enrolment in the community, each participant was invited to a meeting with a study team member and a staff member from COSUP. The meeting occurred at a COSUP site closest to the participant’s place of residence or another convenient and private location with the participant and interviewers present. During this meeting, participants were briefly interviewed to collect post-release data and, if applicable, update location information. Trained field researchers (PN and DM), one male and one female, with prior experience with qualitative research conducted qualitative in-depth interviews at: 30, 60, 120, and 180 days post-release or post-community enrolment. A purposively designed interview guide (supplementary material) that was loosely oriented around the socioecological model [22] to take an ethnographic approach to understand participant’s lives and how substance use intermingled or defined specific elements of their daily experience [23]. As such, the interview guide was a starting point for the interviewers, who developed relationships with the participants over the course of the longitudinal interviews and followed-up on specific conversation elements from prior interviews. Because overdose is common element of hazardous substance use the interview guide for the first interview included a specific question about overdose: “Have you ever overdosed on *nyaope*?” with associated prompts for the interviewers to use if needed to develop the conversation regarding overdose. Subsequent interviews returned to overdose if it was an important aspect of the first interview or if the subject arose when discussing substance use since the last interview. The interviews were conducted in English, Sepedi, or Setswana, based on participant preference. Interviews were audio-recorded and supplemented by field notes. Participants were compensated with the equivalent of USD 6 for each visit. Audio recordings were transcribed verbatim and translated into English (as necessary) by professional transcriptionists and translators. Transcripts were de-identified, and a pseudonym was assigned to each participant using a random name generator. Final transcripts were uploaded to MaxQDA software (VERBI Software, Germany) for analysis.

### Data analysis

The qualitative analysis consisted of a two-phase process. *Phase one* occurred as part of the parent study. All transcripts were coded by two researchers (YA and SP) using *a priori* deductive coding and open coding informed by topics in the interview guide and those that emerged during analysis. This was followed by axial coding to identify relationships between topics or themes. Axial coding occurred iteratively through reading, rereading, memoing, and weekly discussions with the research team including a qualitative expert (JO) and the two qualitative interviewers (PN, DM) to discuss context, themes, and relationships [24,25]. *Phase two* occurred In preparing the manuscript on overdose knowledge and experiences. Two authors with backgrounds in addiction medicine and the local context (UB) and medicine (CJH) and a qualitative researcher (YA) re-read all transcript segments that included overdose content to assure consistency with identified topic areas.

## Results

A total of 23 criminal legal-involved individuals living with HIV and reporting illicit opioid use who were released from incarceration prior to 1 September 2022 and enrolled between 16 September 2021 and 19 May 2022 were included in this analysis. The participants’ median age was 32 years (interquartile range [IQR]: 31, 38). Most self-identified as male (n = 19; 83%), heterosexual (n = 22; 96%), and Black/African (n = 20; 87%) (Table 1). All participants reported two or more incarceration episodes and previous opioid use. Most reported opioid use in the past three months (n = 21; 91%) and injection drug use in the past three months (n = 19; 83%), including during incarceration. A total number of 59 interviews were conducted with the 23 participants with a median duration of 51 minutes (interquartile range: 43, 62) (see Supplementary File for data).

**Table 1:** Characteristics of participants.

Characteristics	n (%)
**Total**	23 (100)
**Sex**	
Male	19 (83)
Female	4 (17)
**Age group**	
20–29	3 (13)
30–39	18 (78)
40–49	2 (9)
**Primary sexual identity**	
Man who has sex with women	19 (83)
Woman who has sex with men	3 (13)
Bisexual woman	1 (4)
**Ethnic group**	
Black/African	20 (87)
White/European	2 (9)
Indian/Asian	1 (4)
**Number of incarceration episodes (including index)**	
≥2	23 (100)
**Last opioid use**	
In the past 3 months	21 (91)
Not in the past 3 months	2 (9)
**Injection drug use**	
Yes, in the past 3 months	19 (83)
Yes, but not in the past 3 months	1 (4)
Never	3 (13)

The researchers organized the findings under two broad domains: knowledge and experience of overdose, and responses to overdose.

### Knowledge and experience of overdose

All participants reported understanding the term “overdose”. However, many were uncertain of how to define it and were often uncertain of the symptoms of opioid overdose. Some participants reflected this uncertainty by framing their answers in the form of a question:

*Overdose as in overdose as in spitting foam or doing what? (Lonnie, male, primarily using nyaope).*

Some participants searched for a way to describe overdose and ended up stating it was excessive use of a drug (e.g., “smoking a lot” or “smoking more than you should”), but without mentioning specific symptoms.

Even among participants who attempted to define or describe overdose, most listed symptoms that did not align with clinical signs of an opioid overdose. Participants most often mentioned fainting/collapsing/falling, foaming at the mouth, and vomiting, rather than symptoms such as irregular or stopped breathing, slow heartbeat, pinpoint pupils, and blue fingernails or lips. As Jewell (male, primarily using methamphetamine) reflected:

*I need to collapse first if it is an overdose… There must be a lefolo (foam) coming out. The foam. It is an overdose thing. Or to faint. That is an overdose.*

One participant specifically described overdose as using more drugs than the body was ready for and identified the period immediately after release from incarceration as a high-risk period for overdose:

*When you come from prison, you are clean, when you come out… isn’t it it’s been a while not smoking, not injecting. When you get outside then you want to inject the whole bag. You are not supposed to inject that whole bag, it will be an overdose for you because you are clean, it’s been a while not smoking. (Adela, female, primarily using nyaope)*

Participants also described vomiting either as their sole symptom, as a symptom that preceded loss of consciousness, or as a symptom that preceded headaches and diarrhea. Rory (male, primarily using *nyaope* and crack cocaine), reported overdosing in prison after using 16–17 bags of drugs, said it was a common experience for many people while incarcerated:

*I used to vomit, when I was in prison… and this other person was also vomiting blood, you see. And I realized that this person no man…, and I even told him that it’s an overdose… yes, a lot, there are many in prison.*

Approximately half of the participants reported having experienced an overdose. Falling or collapsing was the most common symptom that participants reported personally experiencing during an episode. One participant, Lonnie (male, primarily using *nyaope*), described that he “collapse [d for] a whole 3 weeks” after injecting himself with drugs on the day he was released from prison.

Other participants viewed “overdose” through a different lens – not as a negative effect of the drug but as a problem with the individual using the drugs or of the technique of use. For example, Derrick (male, primarily using *nyaope*), viewed overdose-type symptoms as a weakness of the individual experiencing those symptoms:

*We don’t usually call it an overdose… we would say that a person is weak, it [the drug] doesn’t want that person… Maybe the person would have fallen when he was smoking [term used for smoking or injecting nyaope] and then they pass out… Or it kills him… it is not an overdose.*

Although not a widely shared perspective, Derrick’s descriptions placed the responsibility of overdose-like symptoms on the constitution of the person experiencing the symptoms rather than a negative effect from a drug.

Another participant, Wade (male, primarily using *nyaope* and *ntash* (methcathinone)), also described overdose as the fault of the individual. He stated that loss of consciousness – that some may consider an overdose – could result from alternative causes, including from holding one’s breath for too long when injecting in the neck.

Overall, despite reporting some experience and knowledge of overdose, participants struggled to accurately describe symptoms of opioid overdose or ascribed potential overdose symptoms to the individual’s constitution or other injection practices rather than the effects of opioids themselves.

### Responses to overdose

Among the participants who reported experiencing an overdose, few reported receiving medical attention. Those who did receive attention were fortunate enough to have either a family member arrange transport for them to get to a hospital or others to call for an ambulance. Edwin (male, primarily using *nyaope*), who overdosed at home, described being discovered by his family:

*I smoked overdose, and it happened at home, I was injecting in my room. They called an ambulance. There was my mother, my grandmother, and my other cousin… They were watching television. I was in my room. They heard me falling guuu (making a sound of heavy fall). When they came to check, they found that it was me who had fallen.*

However, most participants’ family members were unsure of how to respond to an overdose. The mother of Rory (male, primarily using nyaope and crack cocaine), for example, “did not do anything” even though she became scared after watching her son possibly overdose. Scottie (male, primarily using heroin and crystal methamphetamine), who actively sought his sister’s help when he overdosed at home, was met with confusion and ultimately inaction:

*My sister asked me, what’s wrong, I said I’m gonna die, help me. She said what is it? She threw water at me. She was spitting with water. She said now you better, I said no I’m gonna die still. She then said what must I do [Scottie]? I can’t do anything. She didn’t know what to do. She went out, she went to sleep in another room. She locked the door.*

Most participants also reported overdose events occurring on the streets in the presence of peers. None voluntarily sought medical care. As Stafford (male, primarily using marijuana) recalled:

*I was wet, they poured water on me, others were blowing air on me… I didn’t go to a clinic or hospital… I didn’t go home. I sat there where we were, walked around there. I only went to the house late in the evening to sleep*.

Pouring water onto the overdosing person was the most prevalent response strategy. This was perceived as a reasonable response because overdose was frequently associated with heat or hot blood. As Jack (male, primarily using nyaope and marijuana) explained:

*There are those when they faint, we-we-we pour them with water… Isn’t it when you have smoked overdose, your blood is boiling… So, we pour you with water so that the temperature can go down. We sit you well under the shade so that the temperature wears down and be alright.*

Two participants reported seeing their peers attempt to revive the person who overdosed. They were also the only participants who contacted or watched their peers contact the ambulance. Richard (male, primarily using nyaope), who was present at one of these scenes, noted that the ambulance response time was hours long:

*We were calling the ambulance… he stayed like that for 5-6 hours; he ended up with flies buzzing in his mouth. I thought he was dead whereas he wasn’t. The ambulance came in after 5 to 6 hours and took him… it took him to the hospital…*.

Participants, overwhelmingly stated that they and their peers preferred not to perform *any* action, including touching the person and calling the ambulance because of a fear of potential criminal charges and police involvement by circumstantially being associated with the overdose event and possible death:

*You will touch him, then he dies. Then they will say that it is you. I don’t want to be in that kind of situation. When you have fallen you have fallen, I just watch. (Derrick, male, primarily using nyaope*)

Navigating between their fear of the police and their desire to help, Wade (male, primarily using nyaope and ntash) and his peers decided to relay the incident to others, who then called for help:

*We ran away, what can we do? We told the ladies who are selling that this guy is dying we don’t know what is going on. Those ladies called the police from the nearby police station in Akasia and they came there. When they got there, they found him dead*.

[All names used above are pseudonyms]

## Discussion

The researchers explored how criminal legal-involved people living with HIV and illicit opioid use in South Africa understand and respond to overdose. Although all participants in this study reported familiarity with the term ‘overdose’ and most described experiencing or witnessing an overdose event, their actual understanding of what an opioid overdose was and how to manage it was limited. Participants described a wide range of signs and symptoms, with foaming at the mouth and collapsing being common. The most common “treatment” was pouring cold water onto someone. Importantly, participants identified the period immediately following release from correctional facilities as a key context during which overdoses occurred.

Previous qualitative research suggests that varied understandings of what constitutes an overdose may contribute to a reluctance to seek medical assistance when adverse drug experiences are perceived as insufficiently severe or do not follow expected symptom trajectories [26]. Our findings that an opioid overdose is perceived as heat or foaming may partially explain the reported response of pouring water. Reasons for misperceptions of overdose and limited understanding of management may be a result of the relatively new phenomenon of hazardous opioid use in the studied communities and the absence of training among medical staff in overdose assessment and management.

Inadequate recognition of overdose symptoms can delay or prevent appropriate responses, even in contexts where life-saving interventions such as naloxone are available [27]. It is unclear if our participants faced prolonged delays in overdose assistance. However, the limited understanding of overdose and overdose management are risks for delays. Steps to support safer substance use by equipping people with antidotes to opioid toxicity (e.g., naloxone) depend on the level of recognition of what constitutes an opioid overdose. In other settings, training programs have reduced the knowledge gap and increased bystander willingness to intervene. For example, a study conducted in the United Kingdom demonstrated that training led to significant increases in participants’ ability to recognize and respond to overdose, alongside improvements in their perceived confidence and willingness to administer naloxone [28].

Consistent with studies conducted in North America and South Africa, participants cited fear of law enforcement and stigmatization due to their substance use as primary reasons for not calling emergency medical services when witnessing a suspected overdose [29–33]. This fear may have been particularly heightened due to these participants’ involvement with the criminal legal system; all had been incarcerated more than once, and several were on parole or probation at the time of the interviews. Canada and parts of the United States have enacted drug-related Good Samaritan Laws to encourage people who use drugs to call for emergency medical services by providing some legal protection to overdose victims or bystanders [34]. A study using national data from the U.S. found that Good Samaritan Laws were associated with 16% less risk of opioid overdose death [35].

South Africa currently lacks Good Samaritan Laws related to opioid overdose [36]. While the country’s regulations relating to standards for emergency medical services do not require emergency medical service providers to report drug overdoses to the police, arrests of people providing harm reduction materials and destruction of associated equipment have been documented [37,38]. Policies that protect overdose victims and responders from drug-related arrest, charge, or prosecution coupled with overdose education could be an important step in improving overdose management and reducing overdose morbidity and mortality.

Participants recognized that an increase in overdose risk when returning to the community is potentially related to a decrease in drug tolerance due to limited drug access while incarcerated [13]. Existing literature shows that initiation of opioid agonist or antagonist therapy during incarceration, particularly methadone, significantly reduces post-release opioid-related overdoses [39–41]. At present, services for hazardous opioid use in South African correctional facilities are absent, indicating an opportunity to establish programs in the country [19]. To ensure continued care engagement during re-entry, linkage to community providers should be coordinated prior to release [42,43]. Successful HIV care linkage models during re-entry in South Africa may provide applicable approaches for early implementation [18].

This study highlights the need for overdose response programs among people involved with the criminal legal system and their family members and peers in South Africa. Potential approaches to improve the overdose response may involve education on opioid overdose to people using opioids, training on how to respond, and access to opioid antagonists for the reversal of acute opioid intoxication. In addition, training of health care providers on hazardous opioid use and overdose identification and management could expand services to prevent and manage overdose. The limited understanding of overdose among our participants who had health care contact as PLWH, is an indication of a missed opportunity. We surmise that people living without HIV may have less regular health care contact and fewer opportunities for health care worker initiated discussions on opioid overdose and hazardous opioid use. For PLWH, HIV care visits could serve as an opportunity to assess opioid use and discuss harm reduction and overdose recognition and management.

This study has several limitations. Participants in this study constituted a convenience sample of people returning from incarceration and living with HIV and harmful opioid use and may not be representative of the population overall. The study was undertaken in and around Tshwane, an urban municipality characterized by a high opioid-related burden [37]. As a result, the findings may not reflect perceptions and experiences in other remote or rural settings with fewer treatment options. In addition, we only included PLWH. This may have biased the sample to people with a greater connection to healthcare through HIV testing and treatment services. We also note that biases can arise in participant response including social desirability bias and recall bias. It is possible that social desirability bias caused participants to try to explain overdose even when they were unsure of what it was. We believe that this adds rather than detracts from the findings. Recall bias could have influenced responses to include just the most emotionally charged memories of overdose and response. This also does not distract from the findings, because it provides a view into the most salient beliefs on what constitutes overdose and how to respond.

## Conclusion

This study provides qualitative insight into overdose experience, knowledge, and response among criminal legal-involved people living with HIV and hazardous opioid use in South Africa. Participants displayed varying perceptions of what an overdose is and described common non-medical approaches to address overdoses. Adequate overdose education and training are needed but should be tailored to reflect the local drug market and experience. Future research on intervention development can benefit from collaborations with people who use drugs in South Africa to devise strategies that address the local context. For criminal legal-involved people specifically, correctional facilities may represent an opportune setting in which overdose interventions can be delivered in conjunction with providing medications for opioid use in a coordinated program. To optimize post-release outcomes, policy changes, including instituting legal protection for people who seek or provide medical assistance in the event of an overdose, are needed.

## Supporting information

S1 File Interview guide. (DOCX)

S2 File COREQ checklist. (DOCX)

S3 File Transcript data. (CSV)

## Abbreviations

COSUP: Community Oriented Substance Use Programme
MOUD: Medication for Opioid Use Disorder
PLWH: People Living with HIV
