# Supplementary material for: Knowledge and experiences of overdose among formerly incarcerated people living with HIV and hazardous opioid use in South Africa: A qualitative study
Source: PLoS One. 2026 Nov 2;21(11):e0361907. doi: 10.1371/journal.pone.0361907 (PMC13726910; doi:10.1371/journal.pone.0361907)
Supplement: Supplementary file 1 [file pone.0361907.s001.docx]

**c2c OUD Care**

Reentrant Interview Guides v1.0

There are 4 longitudinal interviews scheduled for 2 weeks post-release and at 2, 4, and 6 months post-release. There is a guide for interview 1 and for interviews 2-4.

**Interview #1: First in-depth interview post release. (This interview should be conducted if no prior interviews have occurred, even if it is not at the first visit date.)**

Personal Background / Typical Day

I would like to begin hearing a little bit about you.

- Can you tell me a little bit about yourself (something that is important that I may not yet know)?
- What is your living situation right now? (key issues: stability and social support)
  - Who are you staying with?
  - How is this good or bad for you right now?
  - is this a situation that will continue for some time?
- How have things have been going now, being back in the community.
  - (for those out for a while: focus on how the transition back into the community has been)
  - What has has gone well? What has been hard?

Social support

- Can you tell me about the people who have been there for you with friendship, information, helping with problems, or other assistance?
  - Tell me more about their support; how do family or friends or others help you?

HIV care experiences

I want to ask about your health care, particularly HIV care.

- Tell me about what you have done to get HIV care after release.
- Tell me about HIV care.
  - How has going to the clinic been?
  - How about remembering to take ARVs?
- How important is getting HIV care / taking ARVs to you compared to all the other things you have to do?
- What things get in the way of your HIV care?

Drug use

- Tell me about your previous experiences with services for people using drugs.
- Have you tried to stop using nyaope (or other substances?)
  - Tell me more about trying to stop (when, why and what did you do)?
  - Tell me about those attempts and what you tried and what happened.
  - (if not already described) Have you ever been in a substance use program before (COSUP)?
- Tell me about drug use in your life – what drugs, when you use, who you use with.

(not all prompts below need to be asked)

- What are your preferred drugs and method of using (Drug of choice / Injection or other)?
- Do you only use that/those drug (s)? What about mixing with other drugs or also drinking alcohol?
- How often do you use?
- Who do/did you use with
- Where do/did you typically use (home, etc)
- Where do/did you get the drugs
- Tell me about how you use the drugs (smoking, injecting, etc.)
- If injecting, where are the needles from?
- Have you used since release – tell me about that.
- Have you ever overdosed on nyaope?
  - What happened?
  - What was the situation when it occurred, where were you, who was around?
  - Describe the help you got
  - Describe the help you didn’t get but would have liked to have gotten
  - What did you do to recover?
  - Have you ever seen someone else overdose? What happened?
- How has nyaope or other opioids (or other drug of choice) affected your life?
  - Tell me about the effect of your use of nyaope on your friends and family.
- How do you manage your HIV care when also using drugs?
- Daily life: medication, drug use, concerns about using drugs while also having HIV
- Whether unable to get to clinic due to drug use
- How do you prioritize drug use versus caring for your HIV health care needs?
- Do providers in AIDS centers know about drug use? How do they treat you?
- Tell me about what’s been happening with your use of drugs since release.
  - When did you last use?

Experience with COSUP

- What did you hope to get from joining COSUP?
- What’s been you experience with COSUP so far.
  - What do you like about COSUP?
  - Has the programme met your expectations? Have your expectations changed?
- Are you still going to COSUP?
  - (If yes,) tell me what keeps you going to COSUP. Can you tell me about a time where you almost didn’t go to COSUP or actually didn’t go? What was the situation
  - (If no,) tell me why have you stopped going to COSUP. What makes it hard to go? Can you tell me some reasons you are not going to COSUP or the clinic? How does going to COSUP or the clinic fit in with your day to day life? What might help you fit them in better?
    - I wanted to ask again if you ever use needles. *(Ask the questions below if yes)*
    - Where do you typically get your needles?
      - Who provides them? How often do you go there? Are they free?
      - Why do you get needles from there?
    - (If needles are not from COSUP)? What about going to COSUP for free needles?
      - What would make it easier for you to get needles from COSUP?
- If you had 3 wishes, what would you want to change? How could substance use treatment fit in?
- What haven’t we asked you today that you think would be valuable for us to know?

NOTE: Continue recording until completely finished with the participant.

**Interview #2-4: These interviews occur at all in depth interview time points after the first interview has been conducted.**

Experiences since release

- Tell me about some good and bad things that have happened to you since we last met.
- Tell me about support you are getting or not getting from family, friends, or others (peer supporters)

Experiences with HIV care

- Have you received any healthcare (gone to the clinic for HIV or other care)?
- (If so) Tell me about healthcare experiences. Tell me about any HIV care you have received or self-care through taking ARVs.
  - (if in HIV care) Do you plan to continue with this care?

(if not in HIV care) what is keeping you from going to clinic and starting ARVs? Experiences with substance use

- Tell me about drug use since we last met.
- Have you used since we last me?
  - If yes, tell me about it. What did you use? How did you use?
  - Have you tried to stop using? Or change the way you use?
    - (if so) what things have you tried?
- What goals (if any) do you have to stop or reduce risks if using drugs
- Have you ever had an overdose since we last met?
  - What happened / what is an overdose like?
  - Did you get help?
  - What did you do to recover?

Experiences with COSUP

- Tell me about COSUP experiences?
  - Describe what has made getting services easy or difficult.
- Are you going to COSUP?
  - If yes, Are they giving you methadone or other medications for substance use?
    - If receiving methadone or buprenorphine: tell me about taking this medication.
      - How helpful is it?
      - What are the downsides of using this medicine?
      - Would you recommend it to others with drug use?
      - What difficulties have you experienced with getting this medication?
        - How could things be made easier
  - (If no,) tell me why have you stopped going to COSUP. What makes it hard to go? Can you tell me some reasons you are not going to COSUP or the clinic? How does going to COSUP or the clinic fit in with your day to day life? What might help you fit them in better?
  - Do you ever use needles?

*(Ask the questions below if yes)*

- Where do you typically get your needles?
  - Who provides them? How often do you go there? Are they free?
  - Why do you get needles from there?
- (If needles are not from COSUP )? What about going to COSUP for free needles?
  - What would make it easier for you to get needles from COSUP?
- What COSUP services have been most helpful?
- How has being in COSUP affected other aspects of your life outside your drug use?
- What additional help would you like from COSUP or your clinic?

Tell me about your substance use plans? Do you think you’ll continue with COSUP?

- If you had 3 wishes related to your healthcare, what would you want to change. How would substance use treatment fit in?
