# Supplementary material for: Knowledge and experiences of overdose among formerly incarcerated people living with HIV and hazardous opioid use in South Africa: A qualitative study
Source: PLoS One. 2026 Nov 2;21(11):e0361907. doi: 10.1371/journal.pone.0361907 (PMC13726910; doi:10.1371/journal.pone.0361907)
Supplement: Supplementary file 2 [file pone.0361907.s002.docx]

COREQ – Overdose Manuscript

| Domain 1 | |  | Page Number |
| --- | --- | --- | --- |
| 1 | Interviewer / Facilitator | PN, DM | 8 |
| 2 | Credentials | Social worker with MPH; researcher with BA |  |
| 3 | Occupation | Field researchers | 8 |
| 4 | Gender | Male, Female | 8 |
| 5 | Experience | training in qualitative interviewing and prior experience conducting qualitative interviews; fluency in language of interviews | 8 |
| 6 | Relationship establishment | Relationship established at study enrollment | 9 |
| 7 | Participant knowledge of the interviewer | No specific knowledge | 6 |
| 8 | Interviewer characteristics | One with lived experience of incarceration, both working with people using substances | 7 |
| Domain 2 | |  |  |
| 9 | Methodical orientation | Grounded theory / ethnography | 8 |
| 10 | Sampling | Convenience | 6 |
| 11 | Method of approach | Face to face recruitment in a correctional facility and in the community | 7 |
| 12 | Sample size | 23 | 9 |
| 13 | Non-participation | Not reported |  |
| 14 | Setting of data collection | Private space in medical building or other location | 8 |
| 15 | Presence of non-participants | Only the qualitative interviewers and participant | 8 |
| 16 | Description of sample | In Results | 9 |
| 17 | Interview guide | Appendix | Appendix |
| 18 | Repeat interviews | Serial interviews at time 0, 1, 3, and 6 months | 8 |
| 19 | Audio/visual recording | Audio recorded | 8 |
| 20 | Field notes | Field notes taken during and after the interview | 8 |
| 21 | Duration | Median 51 minutes | 9 |
| 22 | Data saturation | Assessed during analysis | 8 |
| 23 | Transcripts returned | Transcripts were not returned to participants |  |
| Domain 3 | |  |  |
| 24 | Number of data coders | 2 data coders | 8 |
| 25 | Description of the coding tree |  | 8 |
| 26 | Derivation of themes | Themes developed by 4 people | 8 |
| 27 | Software | MaxQDA | 8 |
| 28 | Participant checking | Participants did not provide feedback |  |
| 29 | Quotations presented | Representative quotations are presented | Results |
| 30 | Data and findings consistent | Yes, data presented reflects the overall findings | Results |
| 31 | Clarity of major themes | Yes | Results |
| 32 | Clarity of minor themes | Yes | Results |
